# Reinforcing Health Data Sharing through Data Democratization

**DOI:** 10.3390/jpm12091380

**Published:** 2022-08-26

**Authors:** Yuhang Wang, Bernd Blobel, Bian Yang

**Affiliations:** 1 Department of Information Security and Communication Technology, Norwegian University of Science and Technology, 2815 Gjøvik, Norway; 2 Medical Faculty, University of Regensburg, 93053 Regensburg, Germany; 3 First Medical Faculty, Charles University, 12800 Prague, Czech Republic; 4 eHealth Competence Center Bavaria, Deggendorf Institute of Technology, 94469 Deggendorf, Germany

**Keywords:** eHealth, data democratization, health data infrastructure, privacy-enhancing technologies

## Abstract

In this paper, we propose a health data sharing infrastructure which aims to empower a democratic health data sharing ecosystem. Our project, named Health Democratization (HD), aims at enabling seamless mobility of health data across trust boundaries through addressing structural and functional challenges of its underlying infrastructure with the core concept of data democratization. A programmatic design of an HD platform was elaborated, followed by an introduction of one of our critical designs—a “reverse onus” mechanism that aims to incentivize creditable data accessing behaviors. This scheme shows a promising prospect of enabling a democratic health data-sharing platform.

## 1. Introduction

Sharing health data creates value for clinical care, trials, and case studies, as well as an improved knowledge base [1,2,3] for healthcare researchers and healthcare organizations. Furthermore, it is crucial for advancing health ecosystems [4]. Health data also have immense commercial value [5] for other parties such as the pharmaceutical industry, data analytics providers, insurers, data markets, or business intelligence. It is also relevant for patients who want to control and share their data (e.g., the service digi.me, the crowd-sourced project wiki.p2pfoundation.net/Category:Health, etc.) in their interests, e.g., monitoring of health status, independent health data analysis, or experience sharing in a patient community.

The huge value associated with health data can lead to data misuse, for example, targeted use of ransomware, participation in the black market [6,7,8], and other cybercrimes. The conventional health data infrastructure was not designed for anticipating value-driven data mobility and the associated cyber threats. There is a structural deficiency in the conventional infrastructure on which patch-like remedies only add to the complexity of the challenge.

Obstacles for health data sharing are data silos, lack of appropriate tooling and lack of needed trust. Rather than reinforcing the infrastructure from a traditional view of vulnerability identification, protection, detection and response, in this project, we address the health data infrastructure’s structural and functional deficiency to facilitate data mobility across trust boundaries through the concept of data democratization and a corresponding set of theories and technologies to implement the concept. Data democratization is a process of making data accessible to everybody and easying the understanding of that data for expediting decision making and supporting the business process [9,10]. Data democratization requires a strong governance for data and process management as well as a related culture, education, training, and tooling to enable this process irrespective of the actors’ domain of expertise and technical know-how. Different contexts and objectives of actors as well as trust antecedents of the actors’ environments establish trust boundaries to be overcome by harmonization/mapping of the related policies [11]. A policy is set of legal, political, organizational, functional, and technical obligations for communication and cooperation, defining the intended behavior of a system [4].

Our work aims at defining, architecting, implementing and evaluating a democratic health data infrastructure that is expected to incentivize all parties, including individuals, to prove, negotiate, and configure their rights and duties associated with health data. The conflicts of interest among different parties can be reconciled through a set of automated mechanisms so that data can be mobilized across trust boundaries.

A burgeoning health data sharing scenario could be more integrated and multifaceted than it used to be. Different parties could have different concerns towards sharing health data, e.g., privacy leakage, technical complexity of interoperability and security, lack of incentives, lack of resources and tools, and high cost of multilateral negotiation. The conventional health data infrastructure is insufficient in coping with data protection in an era of data mobility, e.g., accountability across trust boundaries. Moreover, the plan of future health data infrastructure usually only insufficiently considers fundamental logics and rationales (e.g., risk and incentive modeling, rights negotiation, cognitive modeling, etc.) of data mobility besides technical and regulatory compliance. The complexity attributed to a multitude of social and technical factors makes it difficult to make informed decisions to minimize the risk of a data breach while facilitating data mobility.

We are dedicated to architecting and constructing a data transaction model by strikingly practicing the concept of Data Democratization (or say, democratic data sharing). Formally, this indicates two core ideas, which will be followed throughout the design of our HD platform:

- All stakeholders are treated identically without discrimination. The platform and any constructed protocol would not take into account any player’s distinguishing attributes (e.g., size, market volume, profitability, proprietary technology and knowledge, dominance in administrative power or market influence, information sources, etc.) and therefore each player can be treated equally in our platform;
- The promotion of fairness as a complementary. Based on the first fundamental, this is also essential when facing the inequivalence reality among each party, especially quite often seen between the individual data subject (DS) and the so-called “digital oligarchs”.

State-of-the-art research and ethical and legal efforts have paid extensive attention to the first idea. However, we argue that fairness promotion is also critical concerning data democratization, due to the extremely unequal reality that exists between the individuals and the colossus entity.

For properly responding to the aforementioned challenges and solution weaknesses, we also consider architectural standards as well as related security and privacy specifications from the International Standards Organization (ISO), the European Committee for Standardization (CEN) and Health Level Seven International (HL7). In that context, we have to first mention ISO 23903:2021 [12], the interoperability and integration reference architecture model and framework, but also ISO 22600:2014 [13]. Privilege management and access control, Part 1–3, ISO 21298:2017 [14]. Functional and structural roles, but also the HL7 Privacy and Security Logical Data Model, Release 1, June 2021 [15], all using the ISO 23903 models and principles.

In this paper, we concentrate on a high-level architecture that will meet our design intention. We first provide a brief overview on related works in Section 2. In Section 3, we distinguish different types of stakeholders that would be concerned in our platform based on the differences in motivations, privacy tendencies, functionalities, etc. The definition and description of each derived role are presented in Section 3.1. To improve the universal understanding, in Section 3.2, we provide a mapping between the roles defined in our platform and the roles presented in the European General Data Protection Regulation (GDPR). We deeply consider the hierarchical perspective of our platform and propose, in Section 4, a conceptual architecture to achieve our goal of data democratization. Section 5 illustrates democratic-promoting designs. One of our essential design primitives is a token-based approach to fairness promoting mechanism which focuses on facilitating a “reverse onus” during negotiation between two stakeholders with great disparity. We conclude our work in Section 6.

## 2. Related Works

The national eHealth infrastructure (e.g., local health authorities’ Electronic Medical Record (EMR) systems, north Norway telemedicine infrastructure, and Norsk Helsenett) [16] in Norway has been built since the middle 1990s and is in its design intended for an organization’s internal use, which emphasizes localized data retention and confidentiality. The “one citizen–one journal” plan was proposed in 2012 with the laws regarding medical records and health registers, updated in 2015 to facilitate data mobility. The national pilots Helseplattformen and Helseanalyseplattformen [17] were launched in recent years to technically implement connectivity and coordination in data sharing. On the EU level, the effort has so far mainly focused on the technical (e.g., the epSOS project), policy [18,19,20] and legal [21] interoperability towards the EU eHealth strategy 2020 [22]. As regulations are evolving and national laws always differ, the current health data infrastructure builds segregated silos, differing in purposes, data sharing methods, regulatory compliance practice, and the users’ roles.

The trend towards preventive and personalized healthcare implies that health data can be collected from non-conventional health data sources, such as patients’ devices, living environments [23], and the healthcare industry [24,25,26,27]. These patient-generated health data (PGHD) have frequently not yet been integrated into the national health data infrastructure. We also note that there, so far, exists a trend towards a patient-to-patient [28] crowd-sourced information-sharing community, where the generated new health knowledge may be regarded as PGHD too. However, such platforms are usually plagued by insufficient consideration of privacy.

The data breach caused by health IT outsourcing from Helse Sør-Øst [29] in Norway 2017 received massive public attention. Important concerns have been: the lack of risk management for decision making, lack of diligence from local health authorities regarding data protection of outsourced IT operation, and the lack of technical control (effective rights management in this case). The local municipalities may have an even worse situation [30] due to the fact that they have not adopted the national health data infrastructure. In addition to managerial and technical challenges, we may find it hard to consolidate an unambiguous set of “standard” rules and policies patching up all loopholes or fuzzy zones in laws [31], forcing all organizations and states to unanimous consensus [32]. The complexity attributed to legal, ethical, economic, managerial, interoperability, and technical factors makes security policy and decision making a great burden for all parties dealing with health data mobility, which can be seen from cases such as health record selling [33], data sharing with the government [34] bypassing patients, or patient safety endangered by health data access control in emergency [35,36]. An advanced solution for meeting those challenges is the deployment of ISO 23903 with the ontological representation of policies including ethical ones.

## 3. Classification of Stakeholders and Matching with Roles Defined in GDPR

The prior task for our work is to distinguish discrepant stakeholders with significant behavior characteristics and interest relationships. We first classify our HD platform-relevant stakeholders into seven types. Then, we present a sample of matching between these types of roles and the roles defined in GDPR.

### 3.1. Stakeholders Classification

The HD platform will “circulate” among diverse stakeholders. Some stakeholders participating in the Health Democratization (HD) project intend to get the health data to enable their service provision, while some others have the right of disposal of the health data. Other stakeholders may tend to provide data processing/storage/analyzing facilities. As follows, we classify these stakeholders into sveen different types according to their contexts, objectives and functions, ruled by related policies. A policy is set of legal, political, organizational, functional, and technical obligations for communication and cooperation, defining the intended behavior of a system [4].

- Computing resource manager (CRM)

The service provider assists each actor in managing computing, storage, and communication resources in facilitating data sharing with other actors.

The CRM service is provided through general computing infrastructure layers to support data sharing activities on the logic and operation layers, and is neither intended nor supposed to have any interest in the semantics layer (e.g., the content or utility of the data).

The actor is supposed to fully represent the interest of the stakeholders it serves. Depending on trust models and other factors, one CRM may serve one single or multiple actors. In the latter case, the CRM may have a conflict of interest when it comes to security and privacy aspects.

- Data consumer (DC)

The actor can access data directly, query a database, or receive data from DS, DG, or DSP to exploit the value of the shared data. It is a destination with which the data are shared.

- Data generator (DG)

The actor directly generates data from a DS or converts sensed signals into formatted data, through biomedical sensing, human recording/reporting, social media, human observation, questionnaire, interview, and other technical or non-technical means. A typical DG can be for instance a health or medical sensor, personal mobile device, speech-to-text generator, online questionnaire, a human being, etc.

- Data manager (DM)

The service provider assists each actor in processing, managing, and exchanging the data with other players.

The DM processes, manages, and exchanges data up to the operation layers, and is neither intended nor supposed to have any interest in the semantics layer (e.g., the content or utility of the data). At the syntax level (data structure, data models, database structure, dataset structure, etc.), operations such as formatting, encoding, decoding, transforming, indexing, pseudonymizing, access controlling, encrypting, decrypting, differential privacy-enhancing, content-dependent encryption/decryption, machine learning, data analysis, etc., are included At the binary level (file structure, file management system, etc.), operations such as storing, copying, appending, deleting, encoding, decoding, transmitting, logging, encrypting, decrypting, file format conversion, etc., are included.

DM is supposed to fully represent the interest of the actor/customer served. Depending on applied trust models and other factors, one DM may serve one single or multiple actors. In the latter case, the DM may have a conflict of interest when it comes to security and privacy aspects due to possible trust boundaries.

- Dataset provider (DSP)

The DSP creates and maintains-under the consent given by DS, and possibly the agreement with DG-one or several both syntactically and semantically structured datasets sourced from DS or/and DG, and shares the data with other stakeholders for a data-semantics-dependent purpose consented (in advance or real-time) by DS and harmonized by other involved parties with their rights and obligations. It is a possible source of data provided for sharing. It can also be a destination of shared data.

The DSP differs from DM, as DSP has an interest concerning the content or utility of the data for sharing, while DM does not.

A typical DSP can be: (1) an end dataset provider (e.g., hospital, an Electronic Health Record (EHR) operator, a research institute, etc.); or (2) a proxy dataset provider (e.g., a data portal, a data cache service provider, etc.).

The DSP processes data, not exclusively, on the semantic (content) level, such as appending, deleting, editing, combining, decomposing, transforming, structuring, sanitizing, summarizing, searching, retrieving, anonymizing, content-aware or content-dependent encrypting/decrypting, analyzing, etc., the data.

- Data rights manager (DRM)

The DRM assists each actor in managing his/her rights in relation to other actors, i.e., proving, negotiating, and recording the terms and conditions describing the rights and obligations regarding the data to be shared.

The DRM processes data up to the logic layers and is intended or supposed to have an interest in the data semantics (e.g., the content or utility of the data). This can include activities such as risk and benefit analysis, ethic and socioeconomic constraints, multi-party policy reconciliation, computational strategizing and negotiation, rights and obligations updating and recording, etc.

The DRM is supposed to fully represent the interest of the actor/customer served. Depending on trust models and other factors, one DRM may serve one single or multiple players. In the latter case, the DRM may have a conflict of interest when it comes to security and privacy aspects.

- Data analysis service provider (DASP)

The actor provides data analysis as a service to DS, DG, DC, or DSP.

### 3.2. Relation with Roles Defined in GDPRSubsection

The relation between the participants or stakeholders (DS, DG, DC, DSP, DASP, DRM, DM, and CRM), defined in the Health Democratization (HD) project, and the three roles (“data subject”, “data controller”, and “data processor”), defined in GDPR, is understood in the following way.

The Data Controller is defined in GDPR as the party which determines the purposes and means by which personal data are processed. An organization can be a Joint Data Controller when, together with one or more organizations, it jointly determines ‘why’ and ‘how’ personal data should be processed. Such a joint controller relation must result in an agreement defining the respective responsibilities. The Data Processor processes personal data only on behalf of the controller.

The three roles in GDRP were defined as a legal status to clarify rights and obligations.

The participants in HD are defined in a way taking into account their functional roles in data sharing as well as their interest and rights in the shared data. Thereby, they are defined to facilitate understanding the various data sharing types, models, and scenarios through their independency and dependency relation among each other in function and interest in a specific data sharing transaction.

The data subject defined in HD is equivalent to that in GDPR.

A virtual example for illustrating the relation described above is given as follows:

- **Example**: A general practitioner (GP) can provide a value-added service for his/her patients who have their own Personal Health Record (PHR) system which is technically provided and maintained by a PHR service provider who builds their service on infrastructure provided by the public cloud from Amazon. The GP can specify what data are needed for a health monitoring process for purpose of a specific longitudinal study to personalize the care plan for a specific patient. The GP sets up the longitudinal study plan, collects data from a PHR which has the data sourced from different independent wearable sensor data vault used by the patient, outsources part of the collected data to a data analytics service provider for data analysis purpose, accumulate the data, and finally design a new care plan for the patient. To provide legitimate, auditable, and efficient service information and contract management, the GP uses a contract management App to communicate with the patient for negotiating the rights, obligations, prices, and other issues concerning the offering of the service.

We have developed the following mapping between the aforementioned HD project stakeholder types (GP, patient, PHR service provider, sensor service provider, data analytics service provider) and the GDPR roles, listed in Table 1.

## 4. Conceptual Layered Architecture of HD Platform

To meet the principle of data democratization and the promised capabilities, we gazed deep into the platform from a hierarchical perspective. The HD platform enables developing and managing the democratic negotiation procedures during the healthcare data business, for use in, and exchange of, clinical and individual health information between the potential DS/DM and the potential DC.

For each principle and the potentially promissing scenario, the executive process could be considered as a correlation between the data sharing participants and an affair-related data sharing function at different executive levels, defining the business system’s behavior. The objective must be to adjust the system’s behavior in its structure and function according to the multiple applicable policies from legal, procedural, contextual up to ethical policies and principles including individual policies of the stakeholders involved.

Guided by ISO 23903, which standardized the model and framework of an interoperability and integration reference architecture, but also by ISO 22600 and ISO 21298 (all those standards have also been approved as CEN standards and and re-used in the HL7 security and privacy specifications), as well as the eHealth standardization in the Nordic countries [37] concerning the interoperability [38,39], our HD platform represents the proposed the stakeholders’ classification and related functions. The data sharing function ranges from the incipient data provenance to the rights and obligation tracking according to the agreement. We stratify our platform into four conceptual layers, named “Computing Infrastructure Layer”, “Data Sharing Operation Layer”, “Data Sharing Logic” and “Healthcare Business Layer”. Each layer is eligible for interoperability with its adjacent layers. Our Architecture also obtains references from the peer work on diverse eHealth networking and healthcare data sharing solutions [40,41,42].

**Figure 1:**
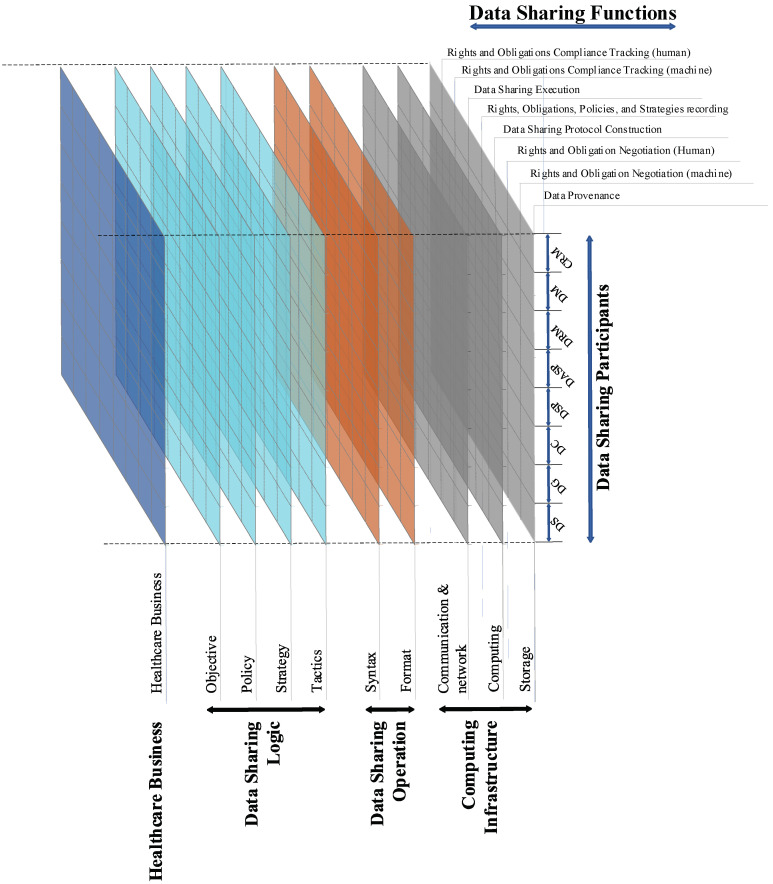
Conceptual Layered Architecture of HD platform.

Figure 1 presents our conceptual architecture in detail. The architectural three-dimensional model describes the data sharing hierarchical structure, the data sharing participants, and the data sharing functions for achieving the business objectives. It outlines a thorough view of related implications of democratic data sharing with our platform. Each square implicates a potential relevance at the practical level. The main systematic-level functions required in our platform include:

- **Data provenance**: providing backward traceability of medical devices, the personal device in the homecare environment, etc., and the health data sourced from these devices to be audited in a trusted way regarding rights and operation status;
- **Risk Assessment**: enabling each data subject to have different risk acceptance tolerance and incentive degrees when they are entitled to rights and benefits from data;
- **Computational negotiation**: negotiating agents can operate and negotiate decisions. The requirements will be developed in compliance with the GDPR, healthcare regulations, and other relevant policies. When processing and exchanging personal data between the agents, the design of the infrastructure will address such key requirements of the GDPR as data protection by design and by default, accountability, pseudonymization, right of access, and right to be informed, to rectify, to erasure, and to be forgotten;
- **Multi-lateral security policing**: enabling individuals to be able to share and control access to health data without having to place extensive trust in entities, and institutions must also be able to share data responsibly for research, innovation, and quality assurance across institutional boundaries.

A dynamic data sharing transaction could consist of the following steps:

- A data provenance process that clarifies among the concerned players the history of the parties with their rights regarding the data to be shared;
- If a default (pre-defined) right and obligation setting is not unanimously agreed upon by the involved players, a knowledge-driven negotiation process must be performed where each player takes into account different factors such as ethical and legal contexts, risk assessment of data breach/privacy breach, benefit from data sharing, etc., based on risk models, and AI-based inference. As business systems are frequently highly dynamic regarding their objectives, context, processes, etc., a dynamic policy management and mapping in consistency with legal and ethical requirements and principles is inevitable;
- The computational negotiation mechanism takes as inputs the risk assessment result from individual players as well as the multi-party security policy logical representation and reconciliation solution, and generates a new recommendation to all the involved parties for achieving an agreement. This process could iterate in several rounds;
- The outcome of the computational negotiation determines the data sharing protocol and the security and privacy-enhancing technical methods for data sharing (e.g., homomorphic encryption, secure multi-party computation, differential privacy methods, federated machine learning, etc.);
- The new configuration of rights of the involved players is recorded using blockchain technology, and the execution of data sharing is encoded into a smart contract which could trigger the automated data sharing now or in the future.

**Figure 2:**
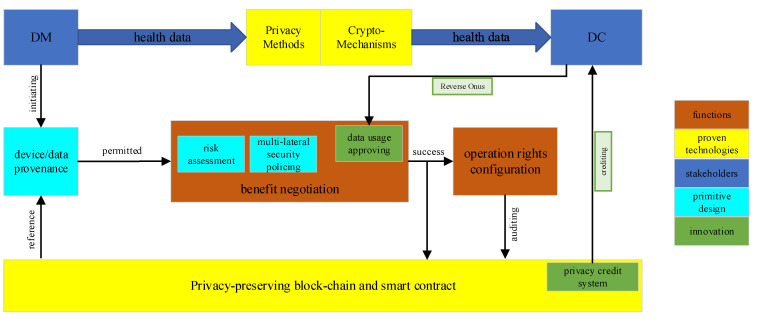
Function-level relational architecture.

The aforementioned design could be merged into a democratic design. Figure 2 shows a function-level relational architecture between the defined roles and the functions.

We illustrated several proven enabling technologies which could be used as a mature solution in the counterpart functions, such as the blockchain-based data provenance mechanism, the conventional privacy-enhancing methods, and crypto-based solutions such as the operable contract enforcement. Our primitive design series for enabling data democratization, such as the risk assessment and multi-lateral security policing, focus on the the negotiating part, which ensures that the backward traceable health data could be traded or shared under an equipotent situation.

In addition, the green part shown in Figure 2 represents one of our innovations, which moves forward to a more democratic vision in the principle of fairness promotion. The next section will provide a brief overview on this part.

## 5. Democratization-Promoting Primitive Design

Numerous state-of-the-art proven technologies and solutions could be utilized for realizing our HD platform. However, a gap still exists between the current solutions and the data democratization vision [43,44]. Our vital task is to design promising technical and procedural solutions that can promote democratic data sharing. For integrating different specifications and solutions, that way enabling comprehensive interoperability, it is inevitable to harmonize the different representation styles and languages by properly re-engineering them on the basis of ISO 23903:2021. Thereby, the axes of the ISO 23903 Reference Model correspond to those in Figure 1 as follows: the ISO 23903 Domain dimension summarizes both the Data Sharing Participants and the Data Sharing Function; the ISO 23903 Development Process dimension is represented by the process-related components; while the ISO 23903 Granularity dimension representing the composition/decomposition of the systems elements is completely missing.

In this section, we will introduce one of the critical data-democratization-promoting designs.

### 5.1. Token-Economy-Powered Incentive Mechanism for Promoting Reverse Onus

In our HD platform, each DC may have the right to claim how much privacy they need to perform a certain healthcare service, whereas the DM may lack the knowledge to assess the validity of the claim. Our HD platform seeks to provide an incentive mechanism to help improve the privacy level of health data. This also could play a role when considering the principle of “data minimization” defined in GDPR.

Considering a vulnerable DS (and his/her DM) with insufficient knowledge to engage in a beneficiary negotiation with the data user, this mechanism will assist this negotiation for achieving a more reasonable scheme or contract from a privacy perspective. It will also cover the execution of the agreement-based contract, especially when the real-world scenario goes beyond the contract’s coverage, by a token-economy-based mechanism and a virtual credit system. The incentive component is expected to restrain the “grey gap” of privacy leakage.

The objectives of the incentive demo include: (1) to provide a “reverse onus” mechanism between data collector and data manager; (2) to promote the faithful execution of the contract; and (3) to inhibit potential incompliant/illegal data user.

In our mechanism shown in Figure 2, the data usage approving helps build the privacy-enhancing consistency between data collector and data manager. After the mutual agreement was achieved and the contract was built, the private credit system will monitor the execution of the protocol to stimulate the data collector to follow the privacy terms, build a token currency system on encouraging privacy-friendly behaviors, and generate the virtual credit of privacy integrity of data collector based on its history log. This credit will be further used to consult the future negotiations.

### 5.2. Data Usage Approval

The negotiation procedure is protected by requiring the data collector to apply form (*appFm*) on the usage of the health data, including:

1. Usage purpose;
2. Data precision upper limit in percentage;
3. Data requesting schedule instant/time period/data manager triggered/etc;
4. Requiring a pattern in the frequency distribution;

The *appFm* will be assessed by the platform, based on the usage log, considering:

1. Purposes to precision. The required data precision should be in accordance with the purposes of the data usage;
2. Purposes to schedule. To assess whether the data accesses conform to the purposes.
3. Purposes to the pattern. To assess whether the data requesting is coherent with the purposes;
4. History comparison across entities.

The *appFm* will always be approved by our incentive component. Here, we only assess the privacy-leakage risk and register the *appFm* in the token currency credit system.

### 5.3. Token Economy Rules

The incentive component organized all the data transmission into a “purchase” behavior in the token-economy system. Here, the component will use a token named “healthcoin”, inherited from our previous work [45], to build a token balance and transaction system. The rules of this token system are as follows:

**Rule 1** (coin creation): For each time the data collector registers the *appFm*, the system will create some healthcoin and transfer them to the data collector’s balance. The amount of the health coin is determined by the details of *appFm* and the credit level of the data collector. By default, for each time the data piece is requested, there will be 1 $ healthcoin generated and transferred to the data collector;

**Rule 2** (health data purchase): Each time a data transmission in the platform happens, the token system will consider it as a purchase behavior of the data collector by using its healthcoin. By default, the price of the health data will be 1 $ as long as it is following the *appFm* claimed by the data collector. The component will always satisfy the purchase if the data collector can afford the price. In combination with **Rule 1**, it is clear that an honest data collector will always work well in our system;

**Rule 3** (credit score): The incentive component will set a credit score for each data collector, denoted as α∈[0, 1], where 1 means data collector has the highest credit score. The credit score will be adjusted based on the simple idea that the more balance of healthcoins it has, the more dangerous the data collector will be, since the credit score will always be satisfied when the data collector has enough healthcoins to obtain whatever data he wants;

**Rule 4** (credit-based coin creation): Based on **Rule 1**, for each *appFm*, the healthcoin DC will gain is (amount × α) $, where the amount is calculated by **Rule 1**;

**Rule 5** (discount): DC can claim a discount by reducing the data requirement (e.g., precision, amount, frequency) to get a discount, the discount strategy is simply following the ratio of data distortion.

### 5.4. Behavior Analysis

An honest and stable DC can be adapted into this system very well, because it always has a low balance, i.e., a high credit score, and earned enough healthcoin for his claimed *appFm*. When a greedy data collector performs:

1. Excessive data transmission, the balance cannot be enough for him to afford the rest of the data, and hence go against his plan of *appFm*;
2. Hoarding the healthcoin (e.g., by utilizing **Rule 5** to save healthcoin on purpose) to perform potential privacy data transmission. However, when the balance becomes high, the gain from the new *appFm* will decrease, and the balance will be exhausted soon since the payments it gets barely cover his expenses.

When an embarrassed DC cannot afford a regular data transmission claimed by himself, he can choose to use the discount to make up for the loss, regain his credit and normal balance in the future.

### 5.5. Incentive Mechanism

Based on the aforementioned token-based mechanism, it will be a choice put in front of DC, which is either to break the balance, be bankrupt, but collect some more health data and then receive profit from it; or to honestly behave as a normal stakeholder, with no gain from extra health data, but also without loss from being bankrupt and leading to harm to the profit from the contract.

The incentive mechanism’s job is to maintain the platform always in a configuration status, which encourage DC to always being the honest part rather than harming DS and DM. Here we use a policy toolkit to configure the global parameters to incentive honest behaviors.

1. The credit parameter

This is the aforementioned parameter to decide how acutely the credit score will decrease with the increase of the healthcoin balance. The incentive mechanism could use this parameter to adjust the balance in the system. For example, when DC finds not enough income and decides to ask for more data, the incentive mechanism can turn up this parameter to achieve a more severe balance reaction;

2. Gain/loss ratio θ

This is a parameter that reflects the gain (from the privacy stealing) and loss (from the regular business). Both gain and loss are inherited from outside information, e.g., the domain expert advice, or the market analysis. Notice that this is not the gain/loss of the virtual healthcoin, but the real-world profit;

3. Discount ratio δ

When the data collector finds it acceptable to discount the data transmission every time to achieve the profit, the incentive mechanism will use discount ratio δ to ensure the discount is no longer cost-effective. For example, obtaining a 50% decrease in data precision with only a 10% discount.

## 6. Conclusions

In this paper, we raised the concept of data democratization, which will reinforce health data-sharing concerning privacy enhancement and benefits insurance. Based on current standards, an overall conceptual layered architecture was proposed which aims to enable such a vision. We illustrated the key components that lead to a democratic data-sharing scenario regarding data provenance, risk assessment, multi-lateral security policing, and computational negotiation. Some proven technologies are also illustrated to cope with the corresponding functions. The output of our HD platform is an executable and auditable contract, democratically signed between well-defined stakeholders. The contract could also be a configuration instruction for conventional privacy-enhancing technologies (e.g., differential privacy) and the crypto-based solutions (e.g., ABE access policies).

We further introduced an advanced concept of data democratization, which emphasized fairness promotion in the HD platform. A token-economy-powered incentive mechanism for promoting “Reverse Onus” on data usage was proposed. This mechanism rebalances inequitable situations among the stakeholders.

Future work will keep on implementing and integrating the proposed conceptual designs. Several landing case studies will be put into consideration to improve the practicability of our work. In that context, we have to harmonize our approach by correctly and comprehensively deploying ISO 23903:2021. ISO 23903:2021 provides a model and framework for a system-theoretical, architecture-centric, ontology-based and policy-driven approach to formally and correctly represent any living or non-living system including its evolution/development [4]. Policies considered ranged from legal, procedural and contextual up to ethical policies and principles. Details will be presented in our paper to pHealth 2022.

## Figures and Tables

**Table 1:** A sample mapping between stakeholders defined above and the GDPR-defined roles.

Party	Participant Defined in HD	Role Defined in GDPR
Patient	data subject	data subject
GP	data consumer	joint data controller
PHR portal managed by the patient	data manager	data processor
PHR service provider	dataset provider	joint data controller, data processor
Amazon cloud	computing resource manager	data processor
Sensor service provider	data generator	data processor
Data analytics service provider	data analytics service provider	data processor
Contract management App	data rights manager	data processor

## Data Availability

Not applicable.
